# MnBa_2_(HPO_4_)_2_(H_2_PO_4_)_2_

**DOI:** 10.1107/S1600536812022775

**Published:** 2012-05-26

**Authors:** Wei Sun, Li-Zhi Sun, Teng-Teng Zhu, Biao-Chun Zhao, Jin-Xiao Mi

**Affiliations:** a Fujian Provincial Key Laboratory of Advanced Materials, Department of Materials Science and Engineering, College of Materials, Xiamen University, Xiamen 361005, Fujian Province, People’s Republic of China

## Abstract

Crystals of manganese(II) dibarium bis­(hydrogenphosphate) bis­(dihydrogenphosphate), MnBa_2_(HPO_4_)_2_(H_2_PO_4_)_2_, were obtained by hydro­thermal synthesis. The title compound is isotypic with its Cd^II^ and Ca^II^ analogues. The structure is built up of an infinite {[Mn(HPO_4_)_2_(H_2_PO_4_)_2_]^4−^}_*n*_ chain running along [100], which consists of alternate MnO_6_ octa­hedra and [PO_4_] tetra­hedra, in which the centrosymmetric MnO_6_ octa­hedra share their four equatorial O-atom corners with tetra­hedral [PO_3_(OH)] groups and their two axial apices with tetra­hedral [PO_2_(OH)_2_] groups. These chains are held together by BaO_9_ coordination polyhedra, developing into a three-dimensional structure. The O—H⋯O hydrogen bonds additionally stabilize the structural set-up. Due to the ionic radius of Mn^2+^ being much smaller than those of Ca^2+^ and Cd^2+^, this may imply that their adopted structure type has a great tolerance for incorporating various ions and the exploitation of more diverse compounds in the future is encouraged.

## Related literature

For background to transition metal phosphates, see: Cheet­ham *et al.* (1999 ▶); Mao *et al.* (2000 ▶); Mi *et al.* (2000 ▶); Sun *et al.* (2012 ▶); Escobal *et al.* (1999 ▶). For isotypic structures, see: Ben Taher *et al.* (2001 ▶) for CdBa_2_(HPO_4_)_2_(H_2_PO_4_)_2_; Ben Tahar *et al.* (1999 ▶) and Toumi *et al.* (1997 ▶) for CaBa_2_(HPO_4_)_2_(H_2_PO_4_)_2_. For the bond-valence method, see: Brown (2002 ▶). For ionic radii, see: Shannon (1976 ▶).

## Experimental

### 

#### Crystal data

MnBa_2_(HPO_4_)_2_(H_2_PO_4_)_2_
*M* *_r_* = 715.55
Monoclinic, 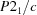

*a* = 5.4168 (10) Å
*b* = 10.1048 (19) Å
*c* = 12.183 (2) Å
β = 100.199 (3)°
*V* = 656.3 (2) Å^3^
*Z* = 2
Mo *K*α radiation
μ = 7.46 mm^−1^
*T* = 173 K
0.25 × 0.22 × 0.08 mm

#### Data collection

Bruker SMART CCD area-detector diffractometer
Absorption correction: multi-scan (*SADABS*; Bruker, 2001 ▶) *T*_min_ = 0.257, *T*_max_ = 0.587
3807 measured reflections
1515 independent reflections
1491 reflections with *I* > 2σ(*I*)
*R*_int_ = 0.021

#### Refinement

*R*[*F* ^2^ > 2σ(*F* ^2^)] = 0.025
*wR*(*F* ^2^) = 0.075
*S* = 1.04
1515 reflections
116 parameters
1 restraint
All H-atom parameters refined
Δρ_max_ = 0.88 e Å^−3^
Δρ_min_ = −1.03 e Å^−3^

### 

Data collection: *SMART* (Bruker, 2001 ▶); cell refinement: *SAINT* (Bruker, 2001 ▶); data reduction: *SAINT*; program(s) used to solve structure: *SHELXS97* (Sheldrick, 2008 ▶); program(s) used to refine structure: *SHELXL97* (Sheldrick, 2008 ▶); molecular graphics: *DIAMOND* (Brandenburg, 2011 ▶) and *ATOMS* (Dowty, 2004 ▶); software used to prepare material for publication: *SHELXL97*.

## Supplementary Material

Crystal structure: contains datablock(s) I, global. DOI: 10.1107/S1600536812022775/br2203sup1.cif

Structure factors: contains datablock(s) I. DOI: 10.1107/S1600536812022775/br2203Isup2.hkl

Additional supplementary materials:  crystallographic information; 3D view; checkCIF report

## Figures and Tables

**Table 1:** Hydrogen-bond geometry (Å, °)

*D*—H⋯*A*	*D*—H	H⋯*A*	*D*⋯*A*	*D*—H⋯*A*
O6—H1⋯O4^i^	0.79 (8)	1.82 (8)	2.600 (4)	172 (7)
O7—H2⋯O5^ii^	0.95 (7)	1.65 (7)	2.504 (4)	149 (6)
O8—H3⋯O2^iii^	0.80 (2)	1.82 (2)	2.623 (4)	178 (8)

Symmetry codes: (i) 
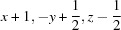
; (ii) 
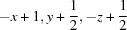
; (iii) 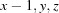.

**Fig. 1.**
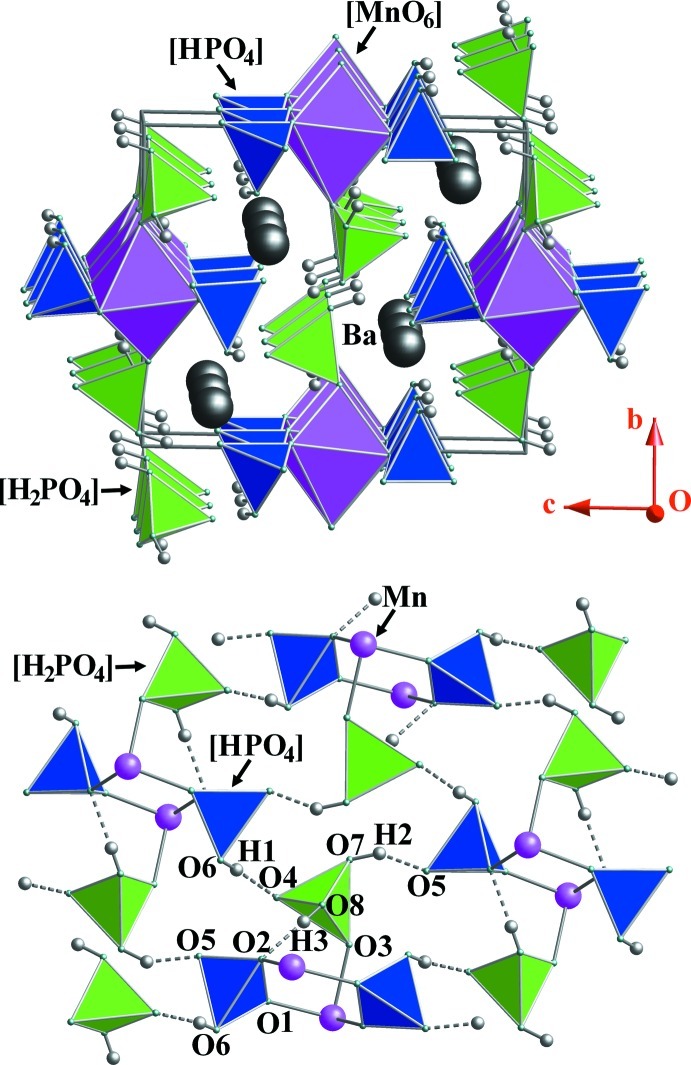
Crystal structure of MnBa2(HPO4)2(H2PO4)2. Upper: viewing down approximately along the direction of [100]. Down: the representation of hydrogen bonds occurring between [PO3(OH)] groups and [PO2(OH)2] groups. ([MnO6] are drawn as pink octahedra; [PO3(OH)] as blue tetrahedra; [PO2(OH)2] as green tetrahedra; Ba as black spheres; Mn as pink spheres; H as small black balls).

**Fig. 2.**
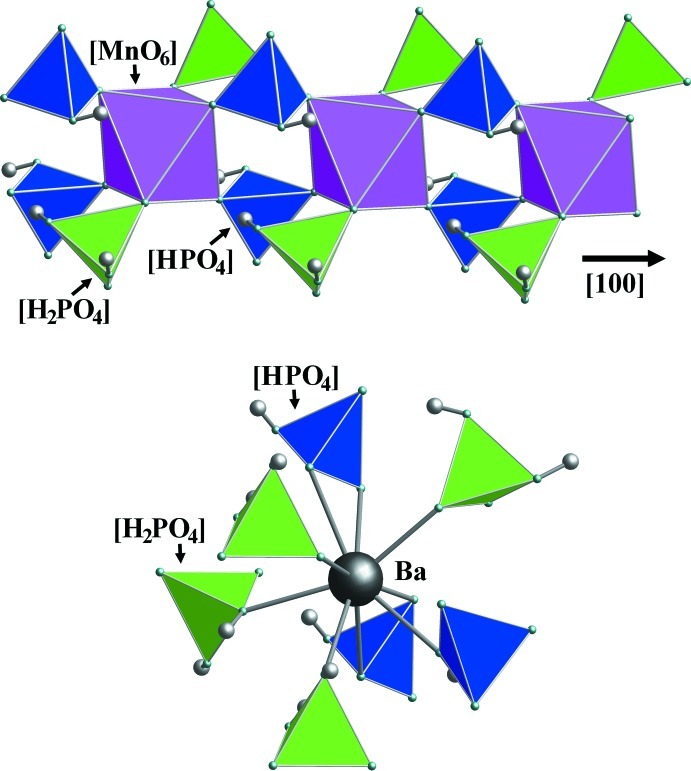
Crystal structure of MnBa2(HPO4)2(H2PO4)2. Upper: the infinite {[Mn(HPO4)2(H2PO4)2]4-}n chain running along the direction of [100]; Down: coordination environment of barium.

**Fig. 3.**
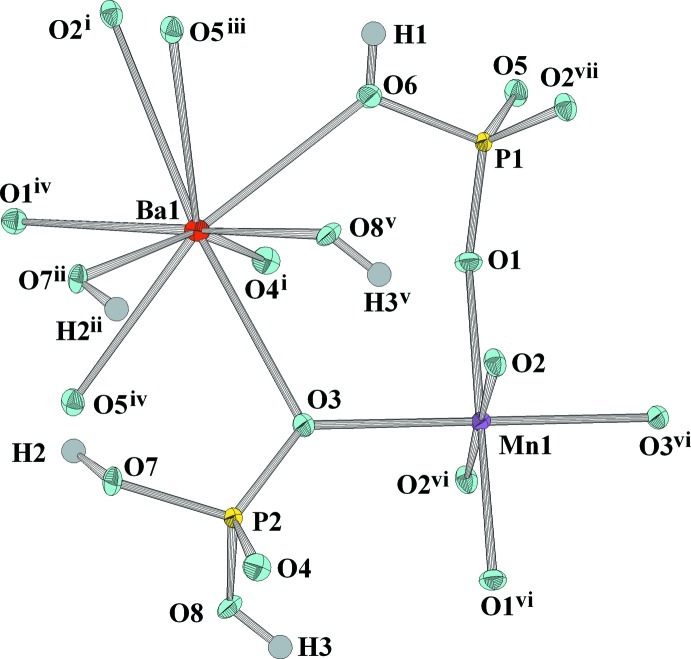
Coordination environment of barium, manganese, and phosphorus atoms, with displacement ellipsoids drawn at the 50% probability level (symmetry codes: (i) x, –y + 1/2, z - 1/2; (ii) –x + 1, –y + 1, –z + 1; (iii) –x + 2, y + 1/2, –z + 1/2; (iv) –x + 1, y + 1/2, –z + 1/2; (v) x + 1, y, z; (vi) –x + 1, –y, –z + 1; (vii) –x + 2, –y, –z + 1)

**Fig. 4.**
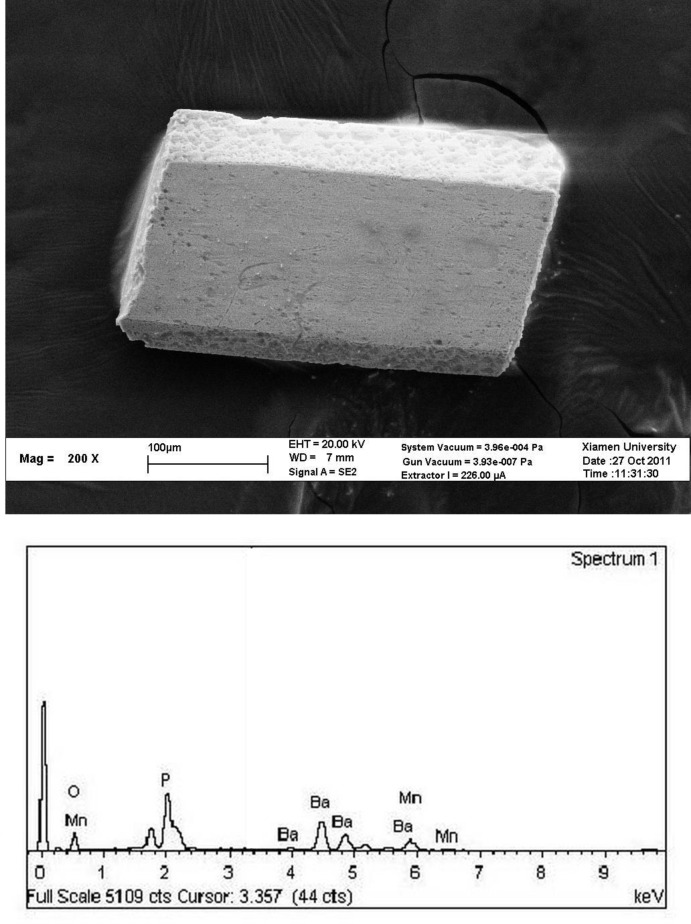
The crystal morphology and chemical composition. Upper: image of scanning electron microscopy for the crystal morphology. Down: the chemical composition of the selected crystal measured by the EDX.

## supplementary crystallographic information

### Comment

Microporous phosphate materials based on transition metal elements have been extensively studied due to their potential optical, electrical, magnetic, and catalytic properties, as well as some interesting properties which are inaccessible to zeolites and other frameworks based on main-group elements only (Cheetham *et al.*, 1999). As a part of our systematical investigation concerning the synthesis and spectroscopic studies of phosphates with transition metal ions, *e.g.* V^3+^, Cr^3+^, Fe^3+^ and Co^2+^*etc*. (Mao *et al.*, 2000; Mi *et al.*, 2000; Sun *et al.*, 2012), herein we report the crystal structure of MnBa_2_(HPO_4_)_2_(H_2_PO_4_)_2_, which has been prepared under hydrothermal conditions. To the best of our knowledge, there is very scant information on manganese barium hydroxy-hydrated phosphates. This is the second hydroxy-hydrated phosphate after the compound of Ba(MnPO_4_)_2_^.^H_2_O (Escobal *et al.*, 1999).

The title compound, MnBa_2_(HPO_4_)_2_(H_2_PO_4_)_2_, is isotypic to its Cd and Ca analogues (Ben Taher *et al.*, 2001; Ben Tahar *et al.*, 1999 & Toumi *et al.*, 1997). In the structure, the asymmetric unit contains one Mn atom, two crystallographically distinct P atoms and one Ba atom (Figs 1–3). Mn1 lies on the inversion center (1/2,0,1/2) (*i.e.* 2*d*) and adopts slightly distorted octahedral coordination with an average bond distance of Mn–O = 2.206 Å. The [MnO_6_] octahedron has six O-corners each fused by corner-sharing to an phosphate tetrahedron, four equatorial O-corners to tetrahedral [PO_3_(OH)] groups and two axial apices to tetrahedral [PO_2_(OH)_2_] groups. P1 is surrounded by three O atoms and one OH group forming a tetrahedral hydrogenphosphate group with an average bond length of P–O = 1.541 Å, while P2 is coordinated to two O atoms and two OH groups forming a tetrahedral dihydrogenphosphate group with *d*(P–O)_av_ = 1.543 Å. Two hydrogenphosphate [PO_3_(OH)] groups bridge pairs of vertices from each octahedron *via* their common O-corners, and *vice versa*, subsequently to develop into a chain running along the direction of [100], and each side of which is decorated by flanking dihydrogenphosphate [PO_2_(OH)_2_] groups (Fig. 2). The infinite {[Mn(HPO_4_)_2_(H_2_PO_4_)_2_]^4-^}_n_ anionic chains are held together by BaO_9_ coordination spheres, which are composed of barium ions (Ba^2+^) surrounded by nine adjacent oxygen atoms with an average bond distance of Ba–O = 2.868 Å, developing into a three-dimensional structure (Figs 1,3) The structural set-up is additionally stabilized by O–H···O hydrogen bonds, which occur between [PO_3_(OH)] and [PO_2_(OH)_2_], and form a two-dimensional hydrogen bonding network parallel to the plane of (100). With regard to the [PO_3_(OH)] groups, one of O-corners bridged to [MnO_6_] octahedra (*i.e.* O2) and one non-bridged O-corner (*i.e.* O5) as well as the OH terminal link individually to their adjacent [PO_2_(OH)_2_] groups *via* hydrogen bonds (*i.e.* 1 OH donor + 2 acceptors). For [PO_2_(OH)_2_] groups, except for O3 connected to a [MnO_6_] octahedron, O4 and two OH terminals link to three [PO_3_(OH)] groups *via* hydrogen bonds (*i.e.* 2 OH donors + 1 acceptor) (Fig. 1). The connectivity of hydrogen bonds is further confirmed by a bond-valence-sum calculation. The bond-valence-sum calculation shows that the bond valence sums of Ba1, Mn1, P1 and P2 are 1.99, 1.96, 4.76 and 4.73 valence units (v.u.), respectively, while those of oxygen atoms O1, O2, O3, O4, O5, O6, O7 and O8 are -1.79, -1.63, -1.81, -1.65, -1.65, -1.36, -1.31 and -1.27 v.u., respectively (Brown, 2002). Oxygen atoms O6, O7 and O8 with the bond valence sums in the ranges of -1.36 to -1.27 v.u. are protonated for compensating the negative charge (*i.e.* of hydroxy oxygen atoms). Oxygen atoms O2, O4 and O5 with the bond valence sums of -1.63 to -1.65 u.v. require a hydrogen bond to compensate the negative charge. This is why among three oxygen atoms (O1, O2, O3) which link to [MnO_6_] octahedra, only O2 is involved in a hydrogen bond. Due to the 6-coordinate effective ionic radius of Mn^2+^ (0.830 Å, HS) is much smaller than those of Ca^2+^(1.00 Å) and Cd^2+^(0.95 Å)(Shannon, 1976), this implies that their adopted structure type has a great tolerance for incorporating various ions and the exploitation on more diverse compounds in the future are encouraged.

### Experimental

The title compound, MnBa_2_(HPO_4_)_2_(H_2_PO_4_)_2_ was synthesized by using a hydrothermal method. Typically a mixture of Ba(NO_3_)_2_ (1.04 g), Mn(CH_3_COO)_2_^.^4H_2_O (0.98 g), KBF_4_ (0.60 g) and H_3_PO_4_ (2 ml) with the molar ratio of Ba:Mn:P=2:2:15 was prepared, and transferred into a Teflon-lined stainless-steel autoclave (30 ml in volume), then heated to and held at 463 K for 3 days. Transparent, light pink crystals of the title compound were obtained by filtration, rinsed with distilled water several times, and dried in desiccators. Optical examination and powder X-ray diffraction (PXRD) analyses were used to identify the phases of the solid products. Scanning electron microscopy was used to document the crystal morphologies (Fig. 4). Chemical compositions of selected crystals were examined by use of an Oxford Instruments Energy Dispersive X-ray Spectrometer (EDX), which confirmed the ratio of Mn:Ba:P=1:2:4 from single-crystal data.

### Refinement

All hydrogen positions were located from the difference Fourier map and tentatively refined without any restraint. Then a common variable was applied for the refinement of isotropic atomic displacement parameters (*U_iso_*) of all hydrogen atoms. After refinement the bond distance of O8–H3 became improper, so soft restraints on both *U_iso_* and the bond length (*d*(O–H) = 0.82 (2) Å) were used for H3 during running the refinement.

### Crystal data

**Table:**

MnBa_2_(HPO_4_)_2_(H_2_PO_4_)_2_	*F*(000) = 662
*M**_r_* = 715.55	*D*_x_ = 3.621 Mg m^−^^3^
Monoclinic, *P*2_1_/*c*	Mo *K*α radiation, λ = 0.71073 Å
Hall symbol: -P 2ybc	Cell parameters from 3807 reflections
*a* = 5.4168 (10) Å	θ = 2.6–28.2°
*b* = 10.1048 (19) Å	µ = 7.46 mm^−^^1^
*c* = 12.183 (2) Å	*T* = 173 K
β = 100.199 (3)°	Prism, light pink
*V* = 656.3 (2) Å^3^	0.25 × 0.22 × 0.08 mm
*Z* = 2	

### Data collection

**Table:**

Bruker SMART CCD area-detector diffractometer	1515 independent reflections
Radiation source: fine-focus sealed tube	1491 reflections with *I* > 2σ(*I*)
Graphite monochromator	*R*_int_ = 0.021
1265 images,φ=0, 90, 180 degree, and Δω=0.3 degree, χ= 54.74 degree scans	θ_max_ = 28.2°, θ_min_ = 2.6°
Absorption correction: multi-scan (*SADABS*; Bruker, 2001)	*h* = −7→7
*T*_min_ = 0.257, *T*_max_ = 0.587	*k* = −13→8
3807 measured reflections	*l* = −15→13

### Refinement

**Table:**

Refinement on *F*^2^	Primary atom site location: structure-invariant direct methods
Least-squares matrix: full	Secondary atom site location: difference Fourier map
*R*[*F*^2^ > 2σ(*F*^2^)] = 0.025	Hydrogen site location: difference Fourier map
*wR*(*F*^2^) = 0.075	All H-atom parameters refined
*S* = 1.04	*w* = 1/[σ^2^(*F*_o_^2^) + (0.053*P*)^2^ + 1.2855*P*] where *P* = (*F*_o_^2^ + 2*F*_c_^2^)/3
1515 reflections	(Δ/σ)_max_ < 0.001
116 parameters	Δρ_max_ = 0.88 e Å^−^^3^
1 restraint	Δρ_min_ = −1.03 e Å^−^^3^

### Special details

**Table:**

Geometry. All e.s.d.'s (except the e.s.d. in the dihedral angle between two l.s. planes) are estimated using the full covariance matrix. The cell e.s.d.'s are taken into account individually in the estimation of e.s.d.'s in distances, angles and torsion angles; correlations between e.s.d.'s in cell parameters are only used when they are defined by crystal symmetry. An approximate (isotropic) treatment of cell e.s.d.'s is used for estimating e.s.d.'s involving l.s. planes.
Refinement. Refinement of *F*^2^ against ALL reflections. The weighted *R*-factor *wR* and goodness of fit *S* are based on *F*^2^, conventional *R*-factors *R* are based on *F*, with *F* set to zero for negative *F*^2^. The threshold expression of *F*^2^ > σ(*F*^2^) is used only for calculating *R*-factors(gt) *etc*. and is not relevant to the choice of reflections for refinement. *R*-factors based on *F*^2^ are statistically about twice as large as those based on *F*, and *R*- factors based on ALL data will be even larger.

### Fractional atomic coordinates and isotropic or equivalent isotropic displacement parameters (Å^2^)

**Table:**

	*x*	*y*	*z*	*U*_iso_*/*U*_eq_	
Ba1	0.63461 (4)	0.35046 (2)	0.322960 (18)	0.00907 (12)	
Mn1	0.5000	0.0000	0.5000	0.00694 (18)	
P1	0.91926 (18)	0.01271 (9)	0.30714 (8)	0.0065 (2)	
P2	0.26314 (18)	0.30069 (10)	0.55243 (8)	0.0072 (2)	
O1	0.6833 (5)	0.0118 (3)	0.3582 (2)	0.0111 (6)	
O2	0.8498 (5)	0.0497 (3)	0.6203 (2)	0.0104 (5)	
O3	0.4241 (5)	0.2178 (3)	0.4898 (2)	0.0098 (5)	
O4	0.3420 (5)	0.3043 (3)	0.6778 (2)	0.0121 (6)	
O5	0.8762 (5)	−0.0571 (3)	0.1943 (2)	0.0104 (6)	
O6	0.9749 (6)	0.1640 (3)	0.2869 (3)	0.0113 (6)	
O7	0.2616 (5)	0.4494 (3)	0.5130 (2)	0.0103 (5)	
O8	−0.0222 (5)	0.2624 (3)	0.5203 (2)	0.0104 (5)	
H1	1.093 (15)	0.168 (6)	0.258 (7)	0.038 (11)*	
H2	0.211 (13)	0.479 (7)	0.439 (6)	0.038 (11)*	
H3	−0.065 (13)	0.197 (5)	0.550 (5)	0.038 (11)*	

### Atomic displacement parameters (Å^2^)

**Table:**

	*U*^11^	*U*^22^	*U*^33^	*U*^12^	*U*^13^	*U*^23^
Ba1	0.00826 (17)	0.01023 (17)	0.00887 (16)	−0.00059 (7)	0.00195 (10)	−0.00110 (7)
Mn1	0.0076 (4)	0.0059 (4)	0.0078 (4)	−0.0006 (3)	0.0029 (3)	−0.0002 (3)
P1	0.0079 (5)	0.0046 (4)	0.0074 (4)	0.0003 (3)	0.0026 (3)	−0.0002 (3)
P2	0.0083 (5)	0.0058 (4)	0.0080 (4)	0.0005 (3)	0.0027 (3)	0.0001 (3)
O1	0.0105 (14)	0.0141 (14)	0.0099 (13)	0.0013 (11)	0.0047 (11)	0.0026 (11)
O2	0.0078 (13)	0.0084 (13)	0.0140 (13)	0.0005 (10)	−0.0007 (10)	−0.0007 (10)
O3	0.0120 (14)	0.0076 (13)	0.0107 (13)	0.0008 (10)	0.0043 (10)	−0.0006 (10)
O4	0.0113 (14)	0.0143 (16)	0.0103 (14)	0.0010 (11)	0.0007 (11)	0.0005 (10)
O5	0.0144 (15)	0.0082 (13)	0.0087 (12)	0.0008 (10)	0.0025 (10)	−0.0018 (10)
O6	0.0145 (16)	0.0090 (13)	0.0120 (14)	−0.0006 (10)	0.0064 (12)	−0.0016 (10)
O7	0.0139 (14)	0.0050 (12)	0.0119 (13)	−0.0005 (10)	0.0016 (11)	−0.0008 (10)
O8	0.0110 (14)	0.0102 (13)	0.0104 (13)	−0.0022 (11)	0.0029 (10)	0.0036 (10)

### Geometric parameters (Å, º)

**Table:**

Ba1—O4^i^	2.663 (3)	Mn1—O2^vi^	2.238 (3)
Ba1—O6	2.726 (3)	Mn1—O3	2.238 (3)
Ba1—O7^ii^	2.829 (3)	Mn1—O3^vi^	2.238 (3)
Ba1—O3	2.837 (3)	P1—O1	1.518 (3)
Ba1—O5^iii^	2.852 (3)	P1—O5	1.526 (3)
Ba1—O5^iv^	2.892 (3)	P1—O2^vii^	1.534 (3)
Ba1—O8^v^	2.906 (3)	P1—O6	1.586 (3)
Ba1—O1^iv^	3.025 (3)	P2—O3	1.510 (3)
Ba1—O2^i^	3.081 (3)	P2—O4	1.512 (3)
Mn1—O1^vi^	2.142 (3)	P2—O8	1.574 (3)
Mn1—O1	2.142 (3)	P2—O7	1.577 (3)
Mn1—O2	2.238 (3)		
			
O4^i^—Ba1—O6	80.03 (9)	O1—Mn1—O3	90.42 (10)
O4^i^—Ba1—O7^ii^	155.43 (9)	O2—Mn1—O3	86.66 (10)
O6—Ba1—O7^ii^	123.55 (9)	O2^vi^—Mn1—O3	93.34 (10)
O4^i^—Ba1—O3	86.04 (8)	O1^vi^—Mn1—O3^vi^	90.42 (10)
O6—Ba1—O3	99.08 (9)	O1—Mn1—O3^vi^	89.58 (10)
O7^ii^—Ba1—O3	83.49 (8)	O2—Mn1—O3^vi^	93.34 (10)
O4^i^—Ba1—O5^iii^	126.50 (8)	O2^vi^—Mn1—O3^vi^	86.66 (10)
O6—Ba1—O5^iii^	63.30 (9)	O3—Mn1—O3^vi^	180.0
O7^ii^—Ba1—O5^iii^	75.33 (8)	O1—P1—O5	111.18 (17)
O3—Ba1—O5^iii^	134.74 (8)	O1—P1—O2^vii^	114.92 (17)
O4^i^—Ba1—O5^iv^	72.07 (9)	O5—P1—O2^vii^	107.95 (16)
O6—Ba1—O5^iv^	151.29 (9)	O1—P1—O6	105.48 (17)
O7^ii^—Ba1—O5^iv^	83.60 (8)	O5—P1—O6	107.96 (17)
O3—Ba1—O5^iv^	72.78 (8)	O2^vii^—P1—O6	109.14 (17)
O5^iii^—Ba1—O5^iv^	141.11 (11)	O3—P2—O4	116.01 (17)
O4^i^—Ba1—O8^v^	126.01 (9)	O3—P2—O8	111.55 (16)
O6—Ba1—O8^v^	64.53 (9)	O4—P2—O8	110.27 (16)
O7^ii^—Ba1—O8^v^	67.45 (8)	O3—P2—O7	110.33 (16)
O3—Ba1—O8^v^	62.73 (8)	O4—P2—O7	105.67 (18)
O5^iii^—Ba1—O8^v^	72.22 (8)	O8—P2—O7	101.92 (15)
O5^iv^—Ba1—O8^v^	128.50 (8)	P1—O1—Mn1	151.07 (19)
O4^i^—Ba1—O1^iv^	68.71 (9)	P1—O1—Ba1^viii^	96.65 (14)
O6—Ba1—O1^iv^	124.66 (9)	Mn1—O1—Ba1^viii^	105.85 (11)
O7^ii^—Ba1—O1^iv^	98.43 (8)	P1^vii^—O2—Mn1	142.65 (17)
O3—Ba1—O1^iv^	121.91 (8)	P1^vii^—O2—Ba1^ix^	93.35 (13)
O5^iii^—Ba1—O1^iv^	100.73 (8)	Mn1—O2—Ba1^ix^	101.64 (10)
O5^iv^—Ba1—O1^iv^	50.17 (8)	P2—O3—Mn1	129.37 (16)
O8^v^—Ba1—O1^iv^	165.22 (8)	P2—O3—Ba1	116.17 (14)
O4^i^—Ba1—O2^i^	85.71 (8)	Mn1—O3—Ba1	114.29 (11)
O6—Ba1—O2^i^	74.60 (8)	P2—O4—Ba1^ix^	133.27 (18)
O7^ii^—Ba1—O2^i^	106.01 (8)	P1—O5—Ba1^x^	102.82 (13)
O3—Ba1—O2^i^	170.38 (8)	P1—O5—Ba1^viii^	101.91 (13)
O5^iii^—Ba1—O2^i^	49.12 (8)	Ba1^x^—O5—Ba1^viii^	141.10 (11)
O5^iv^—Ba1—O2^i^	109.20 (8)	P1—O6—Ba1	119.25 (17)
O8^v^—Ba1—O2^i^	119.07 (8)	P1—O6—H1	108 (5)
O1^iv^—Ba1—O2^i^	59.06 (8)	Ba1—O6—H1	132 (5)
O1^vi^—Mn1—O1	180.0	P2—O7—Ba1^ii^	118.46 (15)
O1^vi^—Mn1—O2	86.79 (11)	P2—O7—H2	125 (4)
O1—Mn1—O2	93.21 (11)	Ba1^ii^—O7—H2	116 (4)
O1^vi^—Mn1—O2^vi^	93.21 (11)	P2—O8—Ba1^xi^	125.90 (14)
O1—Mn1—O2^vi^	86.79 (11)	P2—O8—H3	116 (5)
O2—Mn1—O2^vi^	180.0	Ba1^xi^—O8—H3	116 (5)
O1^vi^—Mn1—O3	89.58 (10)		

Symmetry codes: (i) *x*, −*y*+1/2, *z*−1/2; (ii) −*x*+1, −*y*+1, −*z*+1; (iii) −*x*+2, *y*+1/2, −*z*+1/2; (iv) −*x*+1, *y*+1/2, −*z*+1/2; (v) *x*+1, *y*, *z*; (vi) −*x*+1, −*y*, −*z*+1; (vii) −*x*+2, −*y*, −*z*+1; (viii) −*x*+1, *y*−1/2, −*z*+1/2; (ix) *x*, −*y*+1/2, *z*+1/2; (x) −*x*+2, *y*−1/2, −*z*+1/2; (xi) *x*−1, *y*, *z*.

### Hydrogen-bond geometry (Å, º)

**Table:**

*D*—H···*A*	*D*—H	H···*A*	*D*···*A*	*D*—H···*A*
O6—H1···O4^xii^	0.79 (8)	1.82 (8)	2.600 (4)	172 (7)
O7—H2···O5^iv^	0.95 (7)	1.65 (7)	2.504 (4)	149 (6)
O8—H3···O2^xi^	0.80 (2)	1.82 (2)	2.623 (4)	178 (8)

Symmetry codes: (iv) −*x*+1, *y*+1/2, −*z*+1/2; (xi) *x*−1, *y*, *z*; (xii) *x*+1, −*y*+1/2, *z*−1/2.
